# Transposition Island Pedicle Flap to Repair the Nasal Ala

**DOI:** 10.7759/cureus.66307

**Published:** 2024-08-06

**Authors:** Benjamin R Buttars, Kelley L Turner, Gabriel J Saliba, David B Roy, Michael K Coffin

**Affiliations:** 1 Dermatology, HonorHealth Dermatology Residency, Scottsdale, USA; 2 Medicine, Alabama College of Osteopathic Medicine, Dothan, USA; 3 Medicine, William Carey University College of Osteopathic Medicine, Hattiesburg, USA; 4 Dermatology/Mohs Micrographic Surgery, Pine Belt Dermatology, Hattiesburg, USA

**Keywords:** nasal ala repair, island pedicle flap, alar reconstruction, single-stage procedure, nasal surgery technique

## Abstract

Reconstruction of the nasal ala presents surgical challenges, including loss of the nasofacial junction and vasculature compromise, in addition to achieving a cosmetically satisfactory result. The reconstructive surgeon has a variety of closure techniques to employ, but few allow for acceptable cosmesis in a single-stage procedure. The objective of this study is to discuss a novel approach to alar reconstruction using a melolabial-based transposition island pedicle flap, an alternative to traditional interpolated melolabial flaps and inferiorly based interpolated paranasal flap methods. Our reconstruction method utilizes an island pedicle flap harvested from the nasolabial fold and rotated 165˚ medially and superiorly into a surgical defect on the adjacent ala. The pedicle is placed within the alar facial sulcus for a slight trap-dooring effect, recreating the sulcus. The harvest site is closed linearly, resulting in a fusiform scar line to take advantage of the nasolabial fold. Although delicate care is required while dissecting and positioning the flap, it is an otherwise straightforward procedure. The ideal candidate for this technique presents with loss of the alar subunit with an intact alar rim. The only limitation to this style of flap is that the patient has undergone prior procedures involving the ipsilateral nasolabial fold. The transposition island pedicle flap is a well-tolerated alternative to patient cases that require grafting or more involved multi-step reconstructions to efficiently repair nasal alar defects. This technique provides the patient with a presentable cosmetic result using local tissue with minimal post-surgical complications and alar compromise.

## Introduction

Reconstruction of the nasal ala is performed for skin cancer, defects resulting from trauma, congenital anomalies, or other nasal pathologies. The nasal ala, with its convexities, grooves, and sulci, is one of the more challenging areas for repair [1,2]. Minor errors can create profound aesthetic dissatisfaction [2,3]. This case series demonstrates the use of a 165° rotated island pedicle flap as a single-stage procedure that maintains the structure and function of the affected nasal ala. The interpolated melolabial flap and inferiorly based interpolated paranasal flap remain the more employed options for alar reconstruction [4]. However, both require a two-step approach, taxing resources, time, and recovery. Single-staged melolabial- or nasolabial-based transposition flaps blunt the alar groove yet provide the best donor site for sebaceous skin [1,4]. The melolabial-based transposition island pedicle flap is a hybrid design combining a transposition flap with a traditional triangular island pedicle flap. This single-stage procedure is advantageous in that it utilizes local tissue while maintaining alar integrity.

## Technical report

**Figure 1:**
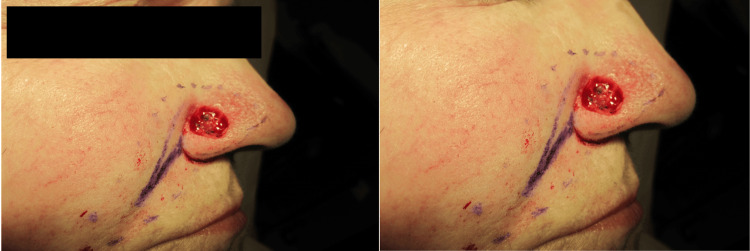
Patient showing loss of the alar subunit

Our variation of the island pedicle uses a medial and superior 165° rotation. The ideal candidate for this flap is a near or complete loss of the alar subunit with sparing of the alar rim (Figure 1). If smaller surgical defects are considered, the entire alar cosmetic subunit should be excised prior to reconstruction to improve cosmesis.

**Figure 2:**
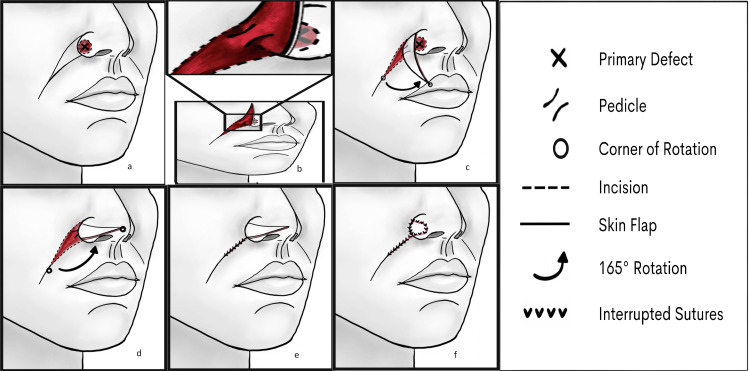
(a) Initial harvest along the nasolabial fold-medial border of the flap congruent with the lateral border of the defect. (b) Pedicle within the alar sulcus. (c, d) Flap rotated medial and superior 165°. (e) Harvest site closed. (f) Flap trimmed and placed to fit the defect Image Credit: Medical Illustrator by Alexis Saliba

The adjacent nasolabial fold is harvested with the medial border of the flap continuous and the lateral edge of the surgical defect occupying the alar facial sulcus (Figure 2a). The width of the flap is excised to match the height of the alar defect. The length of the flap is greater than the width of the alar defect, allowing for medial and lateral tapering of the incision to 30˚ at the caudal end. This eliminates standing cones and provides a transient handle for maneuvering tissue without damaging the flap itself. The length of the tower flap is approximately 2.5 times the length of the defect and should be trimmed to fit once roughly set. This allows a “handle” of redundant tissue with which to manipulate the flap, which will ultimately be removed once in place (38 cases over the last five years).

This is an axial pattern flap, deriving blood supply from the vascular plexus of the facial and angular arteries. The careful undermining of the island pedicle flap begins caudally to the cephalic [5,6]. The flap needs sufficient vascular supply preservation while allowing uncomplicated rotation of the tissue [7]. The pedicle is within the alar facial sulcus to cause a slight trap-dooring effect to recreate the sulcus after precise flap placement (Figure 2b). Suturing should avoid eversion to help with concavity. The mobilized tissue is rotated medially and superiorly 165˚ into the surgical defect (Figure 2c-2d). The harvest site is then undermined widely and closed in a layered linear fashion along the natural nasolabial fold (Figure 2e). The flap is trimmed as necessary to fit the surgical wound and secured in place with a running or interrupted 5-0 nylon suture (Figure 2f). Sutures are removed in seven days.

To avoid nasal valve collapse, we initially utilized a 3 mm-wide cartilage strut to maintain alar convexity harvested from the posterior conchal bowl [8]. Subsequent surgeries have demonstrated that simple nasal packing of the ipsilateral naris for 24 to 48 hours after the procedure can yield similar results. As such, we have abandoned the strut when performing this closure.

## Discussion

There are multiple options when considering reconstruction of the nasal ala. The traditional melolabial flaps cover the defect but have the disadvantages of trapdoor deformities, loss of nasofacial junction, and potential blood supply compromise due to extensive lateral incision. Though Zitelli’s modified single-stage flap significantly improves trapdoor deformity, cosmesis remains a challenge in the nasal ala [9,10]. The rotational island pedicle flap allows for several aesthetically pleasing outcomes. It employs a fusiform scar line, which can be hidden in skin tension lines while maximizing mobility. This minimizes distortion of the surrounding skin [11].

Campbell and Ramsey described a similarly executed transposition island pedicle flap for the reconstruction of nasal and perinasal defects. The authors briefly mention the reconstruction of alar defects in the paper but give no specifics as to the mechanics of the repair. The shark island pedicle flap is yet another option when confronted with an alar defect but may be better suited for defects involving multiple cosmetic units [12].

**Figure 3:**
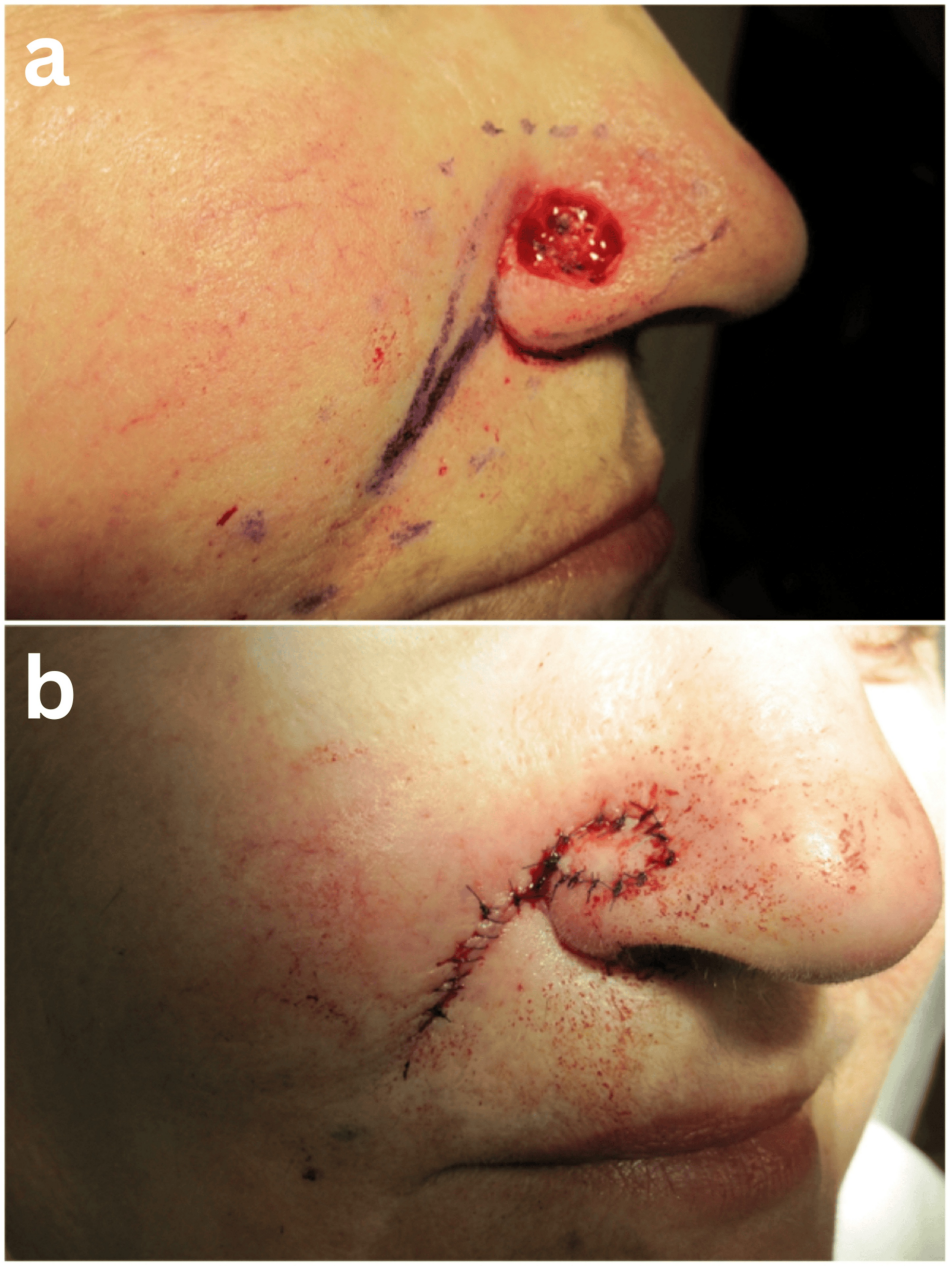
(a) Wound defect of the right nasal ala. (b) Closure of the defect with the island pedicle flap

Potential complications of this variation of island pedicle flap include transient bruising and edema occurring with any surgical reconstruction, nasal valve collapse, necrosis if excess torsion is placed on the vascular stalk, and hypertrophy [3,8]. Improper flap width can also cause distortion due to the elastic recoil highlighted by Zitelli [10]. Excessive flap thickness can occur, though our experience showed that intralesional injection of triamcinolone acetonide was successful in reducing hypertrophy. This flap design is limited only by prior surgeries along the ipsilateral nasolabial fold. Other contraindications common to flaps for alar closures include residual disease, uncertain surgical margins, and previous surgery that may violate the blood supply to the proposed flap. These are avoided due to the single-stage nature of this technique. Figure 3 shows the wound defect of the right nasal ala and the closure of the defect with the island pedicle flap.

This study is limited by the relatively small sample size of 38 cases performed over five years, which may affect the generalizability of the findings. The reliance on a single-stage procedure may not be suitable for all patients, particularly those with extensive prior surgeries or complex nasal defects. The technique described assumes optimal conditions for flap viability and does not account for potential variations in individual healing responses or tissue quality. Additionally, the study does not address long-term outcomes beyond the immediate postoperative period, such as the potential for delayed complications or aesthetic changes over time. This limitation is significant, as minor errors in nasal ala reconstruction can result in profound aesthetic dissatisfaction. The retrospective nature of the study further limits the strength of the conclusions, as does the lack of correlation with patient-specific factors that might influence outcomes.

Recommendations for future studies should include a larger and more diverse patient population to validate the findings and assess the generalizability of the technique. A long-term follow-up is recommended to evaluate the durability of the cosmetic results and monitor for any late-onset complications. Comparative studies with other reconstruction techniques could provide further insights into the relative advantages and limitations of the 165° rotated island pedicle flap. Exploring patient-specific factors that might influence flap success and incorporating objective measures of aesthetic outcomes could enhance the understanding of this approach's effectiveness and reliability. Additionally, addressing the technical challenges, such as ensuring sufficient vascular supply and managing potential variations in tissue quality, will be crucial for improving the outcomes of this procedure.

## Conclusions

We have found superior tissue match in quality, color, and contour, along with a lack of terminal hair transfer, decreased morbidity in single-staged procedures, and preservation of the alar boundaries to be benefits of this technique when compared to other reconstructive options. Adequate closure of the defect with minimal distortion of the local anatomy can be achieved. The use of this island pedicle flap is a tolerated alternative to grafting, transposition flaps, or multi-staged reconstructions for nasal alar defects and consistently affords an excellent cosmetic option with minimal, if any, post-surgical finessing.

## References

[1] Smith H, Elliot T, Vinciullo C (2003). Repair of nasal tip and alar defects using cheek-based 2-stage flaps: an alternative to the median forehead flap. Arch Dermatol.

[2] Cook JL (2009). The lateral ala's volume and position are critical determinants of aesthetically successful nasal reconstruction: a photographic case series. Dermatol Surg.

[3] Mamelak AJ, Wang SQ, Goldberg LH (2009). Linear closure for nasal defects after Mohs micrographic surgery. J Drugs Dermatol.

[4] Fisher GH, Cook JW (2009). The interpolated paranasal flap: a novel and advantageous option for nasal-alar reconstruction. Dermatol Surg.

[5] Dzubow LM (1986). Subcutaneous island pedicle flaps. J Dermatol Surg Oncol.

[6] Otley CC, Roenigk RK (1997). Surgical pearl: preparing the defect for an island pedicle flap. J Am Acad Dermatol.

[7] Chan ST (1988). A technique of undermining a V-Y subcutaneous island flap to maximize advancement. Br J Plast Surg.

[8] Cohen J, Berlin A (2008). Challenge: surgical repair of the alar defect. Skin and Aging.

[9] Carucci JA (2005). Melolabial flap repair in nasal reconstruction. Dermatol Clin.

[10] Zitelli JA (1989). The bilobed flap for nasal reconstruction. Arch Dermatol.

[11] Salmon PJ, Klaassen MF (2004). The rotating island pedicle flap: an aesthetic and functional improvement on the subcutaneous island pedicle flap. Dermatol Surg.

[12] Campbell LB, Ramsey ML (2008). Transposition island pedicle flaps in the reconstruction of nasal and perinasal defects. J Am Acad Dermatol.

